# Hellebrin and its aglycone form hellebrigenin display similar *in vitro* growth inhibitory effects in cancer cells and binding profiles to the alpha subunits of the Na^+^/K^+^-ATPase

**DOI:** 10.1186/1476-4598-12-33

**Published:** 2013-04-26

**Authors:** Laetitia Moreno Y Banuls, Adriana Katz, Walter Miklos, Alessio Cimmino, Daniel M Tal, Elena Ainbinder, Martin Zehl, Ernst Urban, Antonio Evidente, Brigitte Kopp, Walter Berger, Olivier Feron, Steven Karlish, Robert Kiss

**Affiliations:** 1 Laboratoire de Toxicologie; Faculté de Pharmacie; Université Libre de Bruxelles (ULB), Brussels, 1050, Belgium; 2 Department of Biological Chemistry, Weizmann Institute of Science, Rehovot, 76100, Israel; 3 Department of Medicine I, Institute of Cancer Research and Comprehensive Cancer Center, Medical University Vienna, Vienna, Austria; 4 Dipartimento di Scienze Chimiche, Complesso Universitario Monte Sant’Angelo, Università di Napoli Federico II, Napoli, 80126, Italy; 5 Department of Pharmacognosy, University of Vienna, Vienna, 1090, Austria; 6 Department of Medicinal Chemistry, University of Vienna, Vienna, 1090, Austria; 7 Pole of Pharmacology & Therapeutics (UCL-FATH), Angiogenesis & Cancer Research Laboratory, Institut de Recherche Expérimentale et Clinique (IREC), Université catholique de Louvain (UCL), Brussels, Belgium

**Keywords:** Cardiotonic steroids, Cardenolides, Bufadienolides, Cancer, Glycoside / aglycone forms, Lactate release - oxygen consumption

## Abstract

**Background:**

Surface-expressed Na^+^/K^+^-ATPase (NaK) has been suggested to function as a non-canonical cardiotonic steroid-binding receptor that activates multiple signaling cascades, especially in cancer cells. By contrast, the current study establishes a clear correlation between the IC_50_*in vitro* growth inhibitory concentration in human cancer cells and the Ki for the inhibition of activity of purified human α1β1 NaK.

**Methods:**

The *in vitro* growth inhibitory effects of seven cardiac glycosides including five cardenolides (ouabain, digoxin, digitoxin, gitoxin, uzarigenin-rhamnoside, and their respective aglycone forms) and two bufadienolides (gamabufotalin-rhamnoside and hellebrin, and their respective aglycone forms) were determined by means of the MTT colorimetric assay and hellebrigenin-induced cytotoxic effects were visualized by means of quantitative videomicroscopy. The binding affinity of ten of the 14 compounds under study was determined with respect to human α1β1, α2β1 and α3β1 NaK complexes. Lactate releases and oxygen consumption rates were also determined in cancer cells treated with these various cardiac glycosides.

**Results:**

Although cardiotonic steroid aglycones usually display weaker binding affinity and *in vitro* anticancer activity than the corresponding glycoside, the current study demonstrates that the hellebrin / hellebrigenin pair is at odds with respect to this rule. In addition, while some cardiac steroid glycosides (e.g., digoxin), but not the aglycones, display a higher binding affinity for the α2β1 and α3β1 than for the α1β1 complex, both hellebrin and its aglycone hellebrigenin display ~2-fold higher binding affinity for α1β1 than for the α2β1 and α3β1 complexes. Finally, the current study highlights a common feature for all cardiotonic steroids analyzed here, namely a dramatic reduction in the oxygen consumption rate in cardenolide- and bufadienolide-treated cells, reflecting a direct impact on mitochondrial oxidative phosphorylation.

**Conclusions:**

Altogether, these data show that the binding affinity of the bufadienolides and cardenolides under study is usually higher for the α2β1 and α3β1 than for the α1β1 NaK complex, excepted for hellebrin and its aglycone form, hellebrigenin, with hellebrigenin being as potent as hellebrin in inhibiting *in vitro* cancer cell growth.

## Background

A large proportion of cancer patients fail to respond to conventional cytotoxic chemotherapy because of the intrinsic resistance of cancer to pro-apoptotic stimuli and/or the acquisition of multidrug resistance (MDR) during chronic treatment. As emphasized by Pardo et al. [1], the concept of ion channels and pumps as cancer targets has recently gained considerable attention, and the Na^+^/K^+^-ATPase (the Na/K pump, i.e., NaK) could be targeted to combat chemoresistant cancers [2-4].

NaK is composed of α and β subunits. Four α and three β subunits have been cloned with distinct tissue-specific distribution and physiological functions [2-5]. The Na/K pump maintains the concentration gradients of Na^+^ and K^+^ ions across the surface membrane of animal cells by exporting 3 Na^+^ ions and importing 2 K^+^ ions at the expense of hydrolysis of a single ATP up to 100 times each second [6]. However, a substantial amount of surface-expressed NaK in certain types of cells has been suggested to function as non-canonical cardiotonic steroid-binding receptors [7] that form complexes with caveolin-1, Src kinase and epidermal growth factor receptor (EGFR) to activate multiple signaling cascades [8-11] that are markedly different between normal and cancer cells [2-4].

**Figure 1:**
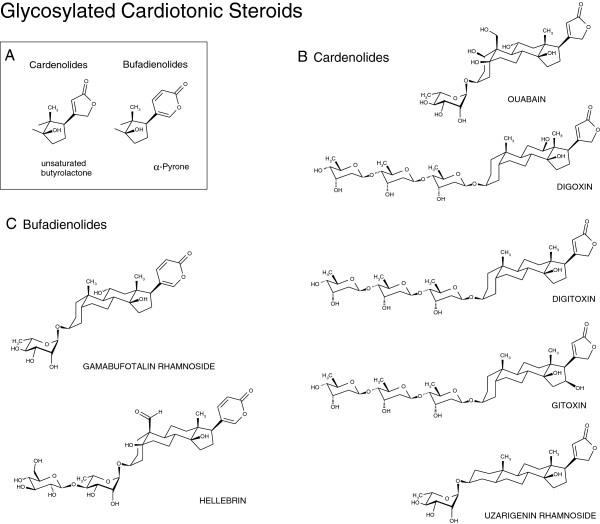
**Chemical structures of the cardiotonic steroids under study. A:** The characteristic unsaturated butyrolactone and alpha-pyrone substructure of cardenolides and bufadienolides, respectively. The individual glycosylated **B:** cardenolides and **C:** bufadienolides under study differ mainly in the hydroxylation pattern and the nature of the sugar moiety.

The most potent and selective NaK ligands, which bind to NaK α subunits, are cardiotonic steroids represented by two classes of compounds known as the cardenolides (including digoxin and ouabain; Figure 1) and the bufadienolides (including hellebrin; Figure 1) [2-5].

Digoxin has been used for decades to treat heart failure due to its ability to increase the force of contraction (inotropic effect) and reduce heart rate, but digoxin remains a dangerous drug because it has a narrow therapeutic window, and can lead to cardiac arrhythmias [2-5]. Indeed, excessive NaK inhibition by digoxin can cause calcium overload, which can in turn cause arrhythmias [5]. The α2 isoform plays a more important role in calcium handling in cardiac contraction compared to the α1 and α3 isoforms, and digoxin displays moderately higher selectivity for the α2 and α3 isoforms over the α1 isoform [5,10,12].

Digoxin has also been shown to have significant therapeutic benefits in breast [13,14] and prostate [15] cancers. However, the levels of expression of the various α subunits have not been determined in breast and prostate cancers. The NaK α1 subunit is overexpressed in a significant proportion of cases in melanomas [16], kidney cancers [17], non-small-cell lung cancers (NSCLCs) [12] and glioblastomas [10]. In contrast, the NaK α3 subunit is overexpressed in a significant proportion of colon cancers [18] and hepatocellular carcinomas [19]. Thus, while digoxin seems to be associated with promising anticancer effects in breast [13,14] and prostate [15] cancers, the full anti-cancer potential of this drug has not yet been addressed. It is necessary to analyze digoxin in the cohorts of cancer patients enriched with the α3 isoform because of the preferential binding of digoxin to the NaK α3 subunit over the α1 subunit [5,10,12]. In contrast, more selective NaK α1 ligands are required to combat those melanomas, glioblastomas, kidney cancers and NSCLCs that overexpress the NaK α1 subunit. We show here that hellebrin and, more surprisingly, its deglycosylated form, hellebrigenin, display distinct *in vitro* anticancer effects and NaK α-subunit-binding patterns when compared to digoxin and other cardiotonic steroids. The present study also shows that gamabufotalin-rhamnoside displays more powerful *in vitro* anticancer activity than any other cardiotonic steroids under study, including conventional cardenolides such as ouabain, digoxin and digitoxin.

## Materials and methods

### Compounds

Ouabain (O3125), ouabagenin (O2627), digoxin (D6003), digoxigenin (D9026), digitoxin (D5878) and gitoxigenin (G4635) were obtained from Sigma-Aldrich NV/SA (Bornem; Belgium). Hellebrin was isolated (at the *Dipartimento di Scienze Chimiche, Università di Napoli Federico II*, Italy) from *Helleborus purpurascens* according to a modified procedure from Cioaca and Cucu [20]. Gitoxin (ASB-00007232-005) was obtained from ChromaDex Inc. (Miami, FL). Uzarigenin-rhamnoside was isolated from *Roupellina* (*Strophanthus*) *boivinii* according to a procedure described by Karkare et al. [21], and was a gift from Prof. W. Schoner (Univ. Giessen, Germany). In order to verify the structure of the compound it was characterized at the Weizmann Institute by ^1^H- and ^13^C- NMR and High Definition, Q-TOF Mass Spectrometric analysis. Uzarigenin was obtained from Chemos Gmbh (Regenstauf, Germany). Hellebrigenin was obtained from hellebrin hydrolysis (Department of Pharmacognosy; University of Vienna, Austria). Gamabufotalin-rhamnoside was isolated from different *Urginea* species [22-24]; gamabufotalin was isolated from toad venom of *Bufo melanostictus*[25]; both were structure elucidated by B.K. (Department of Pharmacognosy, University of Vienna). All compounds under study were obtained with a purity > 95%. All compounds were prepared in a 10^-2^M DMSO stock solution and then diluted in PBS or water to carry out the experiments.

### Cancer cell lines

**Figure 2:**
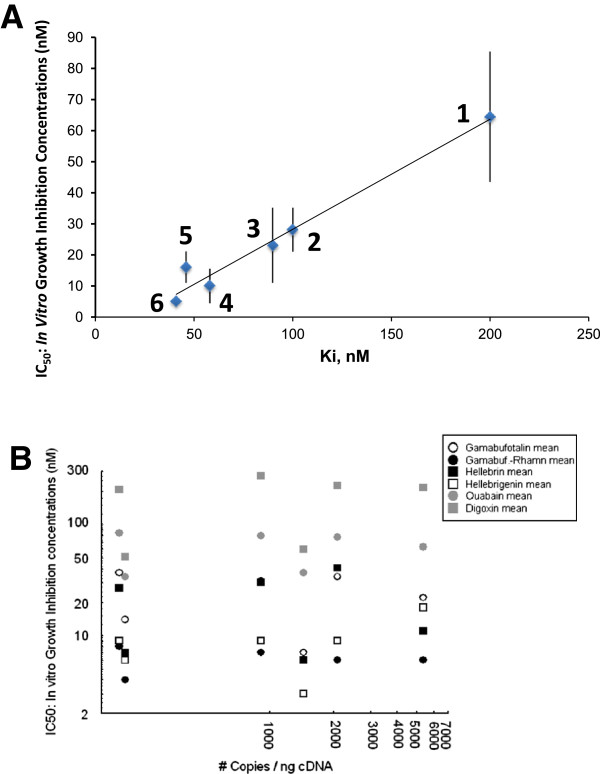
**NaK alpha1-subunit and in vitro growth inhibition of cancer cells. A:** Correlation of Ki for inhibition of human α1β1 by cardiac glycosides and the growth inhibition effects (IC_50_) of human cancer cells; 1: digoxin; 2: hellebrin; 3: ouabain; 4: oleandrin; 5: hellebrigenin; 6: gamabufotalin-rhamnoside. IC_50_ values are the averages ± SEM of 4 experiments with 10 different human cancer cell lines from the NCI60 library: ACHN (renal cell carcinoma); SF-268 and SNB-75 (glioma); MCF-7 (breast cancer); SKMEL-5 (melanoma); HCT-116 and HT-29 (colon cancer); A549 (NSCLC); TK-10 (kidney cancer); and Ovcar-3 and Ovcar-4 (ovarian cancer). In this experiment, the IC_50_ values were determined after two days of culture using the crystal violet assay [54]. **B:** Illustration of the IC_50_*in vitro* growth inhibitory concentration (MTT colorimetric assay; Y axis) as opposed to the mRNA levels (by means of quantitative RT-PCR as detailed in [10]) of the NaK α1 subunit in five human cancer cell lines, including Hs683 oligodendroglioma (207 mRNA copies / μg cDNA); T98G GBM (911 mRNA copies / μg cDNA); A549 NSCLC (1,450 mRNA copies / μg cDNA); U373 GBM (2,091 mRNA copies / μg cDNA); and PC-3 prostate adenocarcinoma (5,337 mRNA copies / μg cDNA) cells. The IC_50_ growth inhibitory concentrations that are reported in Figure 2B are from Table 1.

The histological types and origins of the eight human cancer cell lines that were used for the MTT colorimetric assay are detailed in the legend of Table 1. Two mouse cancer cell lines were also used, the CT26.WT colon cancer cell line (ATCC code CRL-2638) and the B16F10 melanoma cell line (ATCC code CRL-6475). Both cell lines were obtained from the American Type Culture Collection (ATCC, Manassas, VA). A control cell line (human NHDF fibroblasts) was obtained from PromoCell (code c-12300; Heidelberg, Germany). The cell lines detailed in Figure 2A are a generous gift from the National Cancer Institute (NCI, Bethesda, USA) to Steven Karlish’s lab.

**Table 1:** ***In vitro *growth inhibitory concentrations at 50% (IC**_**50**_**) in human cancer cells after three days of culture in the presence of the drug of interest**

**Compounds**	**IC**_**50 **_**(nM)**								
	**A549**	**U373**	**Hs683**	**T98G**	**MCF-7**	**SKMEL-28**	**PC-3**	**HT-29**	**Mean ± SEM**
**Cardenolides**									
Ouabain*	37 ± 2	78 ± 1	34 ± 3	80 ± 3	100 ± 17	83 ± 13	63 ± 10	84 ± 8	70 ± 8
Ouabagenin*	866 ± 25	5,281 ± 250	1,902 ± 167	3,600 ± 36	4,011 ± 202	2,563 ± 183	2,708 ± 62	3,482 ± 19	3,052 ± 479
Digoxin*	60 ± 7	227 ± 42	51 ± 6	274 ± 17	363 ± 35	220 ± 26	215 ± 71	208 ± 20	202 ± 37
Digoxigenin*	395 ± 34	3,188 ± 129	794 ± 22	1,171 ± 99	5,321 ± 170	4,005 ± 116	3,880 ± 87	4,458 ± 83	2,902 ± 658
Digitoxin**	11	61	15	32	187	59	37	198	75 ± 26
Digitoxigenin**	92	369	79	166	2,134	2,495	298	230	733 ± 349
Gitoxin**	68	351	79	174	828	679	361	352	362 ± 96
Gitoxigenin**	1,715	4,477	653	3,589	5,193	> 10,000	3,964	4,403	> 4,249
Uzarigenin-rhamnoside**	45	247	38	38	469	222	77	41	147 ± 55
Uzarigenin**	3,169	7,918	2,252	5,138	> 10,000	> 10,000	6,281	6,439	> 6,025
**Bufadienolides**									
Gamabufotalin-rhamnoside*	3 ± 1	6 ± 2	4 ± 1	7 ± 1	17 ± 5	18 ± 7	6 ± 1	8 ± 1	9 ± 2
Gamabufotalin*	7 ± 1	34 ± 1	14 ± 3	31 ± 8	40 ± 7	22 ± 1	22 ± 4	37 ± 3	26 ± 4
Hellebrin*	6 ± 1	41 ± 2	7 ± 1	30 ± 1	58 ± 12	40 ± 5	11 ± 1	27 ± 4	28 ± 7
Hellebrigenin*	3 ± 1	9 ± 1	6 ± 1	9 ± 1	42 ± 2	28 ± 9	18 ± 1	9 ± 1	16 ± 5

* the data were obtained from three independent experiments, with all experiments carried out in six replicates.

** the data were obtained from one experiment carried out in six replicates.

The human cancer cell lines were obtained from the American Type Culture Collection (ATCC, Manassas, VA), the European Collection of Cell Culture (ECACC, Salisbury, UK), and the Deutsche Sammlung von Mikroorganismen und Zellkulturen (DSMZ, Braunschweig, Germany). The analyzed cell lines include the A549 NSCLC (DSMZ code ACC107); the U373 (ECACC code 89081403), T98G (ATCC code CRL-1690) and Hs683 (ATCC code HTB-138) glioma; the SKMEL-28 melanoma (ATCC code HTB-72); the MCF-7 breast (DSMZ code ACC115); the PC-3 prostate (DSMZ code ACC465); and the HT-29 colon (ATCC code HTB-38) cancer models.

### Determination of in vitro growth inhibitory activity

The cancer cells were cultured in RPMI (Lonza, Verviers, Belgium) medium supplemented with 10% heat-inactivated fetal calf serum (Lonza). All culture media were supplemented with 4 mM glutamine, 100 μg/mL gentamicin, 200 U/mL penicillin and 200 μg/mL streptomycin (Lonza). The NHDF fibroblasts were cultured in Lonza medium cc3132 KT FGM-2 BulletKit.

The overall growth level of the human cancer cell lines was determined using a colorimetric MTT (3-(4,5-dimethylthiazol-2yl)-2,5-diphenyltetrazolium bromide, Sigma, Belgium) assay as detailed previously [10,12,16]. Briefly, this test measures the number of metabolically active (thus living) cells that are able to transform the yellow MTT into the blue formazan dye via a mitochondrial reduction involving succinate dehydrogenase. The amount of formazan obtained at the end of the experiment (measured by spectrophotometry) is directly proportional to the number of living cells. The determination of the optical density in the control compared to the treated cells therefore enables quantitative measurements of the effects of compounds on the growth of normal as well as cancer cells *in vitro*.

Each experimental condition was performed in six replicates.

### MDR cancer cell lines

The following human cancer cell lines and their chemoresistant sublines were used in this study: the colon carcinoma cell line HCT-116 p53/wt and the p53 knock-out cell line HCT-116 p53/ko (generously donated by B. Vogelstein, Johns Hopkins University, Baltimore, MD, USA); the epidermal carcinoma-derived cell line KB-3-1 and the ABCB1-overexpressing subline KB-C-1 (generously donated by D.W. Shen, Bethesda, USA); the small cell lung carcinoma cell line GLC-4 and the ABCC1- and LRP-overexpressing subline GLC-4/ADR (from E.G. de Vries, Groningen, Netherlands); the ovarian carcinoma cell line A2780 and the cisplatin resistant subline A2780cis (purchased from Sigma-Aldrich); and the promyelocytic leukemia cell line HL60 and the mitoxantrone resistant subline HL60/mx (generously donated by G. Harker, Salt Lake City, USA). All cell lines were grown in RPMI 1640 supplemented with 10% fetal bovine serum with the exception of the HCT-116 cells, which were grown in McCoy’s medium with 10% serum.

### Binding affinity of cardiotonic steroids, inhibition of activity of NaK isoforms

Expression of the human isoforms α1β1, α2β1 and α3β1 in *Pichia pastoris* (strain SMD1165) and purification of the detergent-soluble isoform proteins, ^3^H-ouabain-binding competitive displacement by other cardiotonic steroids on *P. pastoris* membranes expressing human α1β1, α2β1, and α3β1 isoforms, and analysis of the binding data was performed as previously described [5]. ^3^H-ouabain binding to yeast membranes (200–300 μg protein) was assayed at 37°C for 1 hour in a medium containing MOPS-Tris 10 mM, pH 7.2; MgCl_2_, 3 mM; Vanadate-Tris, 1 mM; EGTA-Tris, 1 mM [26]. Binding of ouabain or competitive displacement by other cardiac glycosides was assessed by varying total concentrations of ouabain or other cardiac glycosides at constant ^3^H-ouabain (between 1-2 nM (specific activity) 30–40 Ci/mmol). K_0.5_ was calculated using a one site inhibition model: B/B_CG=0_ = K_0.5_/([CG] + K_0.5_). B refers to the ^3^H-ouabain bound at a particular concentration of the cardiac glycoside [CG] and B_CG=0_ refers to the ^3^H-ouabain bound at 1-2nM ^3^H-ouabain in the absence of other cardiac glycosides. The K_D_ was calculated from K_0.5_ by taking into account ouabain-CG competition as K_D_ = K_0.5_/ (1 + [Ou_f_]/ K_DOu_). With K_DOu_ values α1β1 9.2 nM, α2β1 21.5 nM and α3β1 11 nM, respectively [5]. At 1nM total ouabain, the values of 1 + [Ou_f_]/ K_DOu_ were 1.06, 1.03, and 1.06, respectively. Binding of each cardiac glycoside was estimated in 3 separate experiments.

The inhibition of NaK activity of the purified detergent-soluble α1β1, α2β1, and α3β1 complexes by cardiotonic steroids and an analysis of the inhibition data (Ki values) were also performed as previously described [5]. The inhibitors were added to the recombinant enzyme (0.08–0.2 μg of protein) in 400 μl of reaction medium containing 130 mM NaCl, 5 mM KCl, 3 mM MgCl_2_, 25 mM histidine, pH 7.4, 1 mM EGTA, 0.01 mg/ml SOPS, 0.001 mg/ml cholesterol, and 0.005 mg/ml C_12_ E_8_ in 48-well plates. The reaction (37°C for 1 h) was started by the addition of 1mM ATP. Pi release was measured with a malachite green dye to detect the phosphor-molybdate (Pi Color Lock, Innova Biosciences). The percent inhibition V /V_0_ was calculated for each cardiac glycoside concentration, and Ki values were obtained by fitting the data to the function V /V_0_ **=** Ki /([CG] **+** Ki ) (using Kaleidagraph). V_0_ and V represent the control rate and rate of NaK-ATPase activity at particular concentrations of cardiac glycosides, [CG], respectively.

Average K_D_ or Ki values ± SEM for each isoform were calculated. Statistical significance was calculated by the unpaired Students t-test. P values <0.05 were considered significant.

### Lactate release and O_2_ consumption rate

For enzymatic determination of lactate production, confluent tumor cells were incubated with 10 mM glucose for 24 hours. Culture medium was then filtered through centrifugation columns with a 10 KDa cutoff, and L-lactate concentrations were determined on an ISCUSflex analyzer (CMA Microdialysis AB, Solna, Sweden). O_2_ consumption was determined using the MitoXpress assay according to the manufacturer’s instructions (LuxCell Biosciences, Cork, Ireland). Briefly, after adding the oxygen probe to tumor cells cultured for 24 hours in 96-well microplates with or without the tested compounds, the wells were sealed with mineral oil, and respiration was evaluated using time-resolved fluorescent plate readers. Under these conditions, O_2_ depletion was translated into an increase in probe phosphorescence signal, and the slope of this signal was used to derive the O_2_ consumption rate.

## Results

### In vitro growth inhibitory effects of glycosylated versus non-glycosylated forms of cardenolides and bufadienolides in human cancer cells

In general, cardenolides and bufadienolides share a steroid backbone, but these compounds differ from each other on the basis of an unsaturated butyrolactone (cardenolides) versus an α-pyrone (bufadienolides) moiety (Figure 1A). The chemical structures of the cardenolides and bufadienolides under study are illustrated in Figures 1B and 1C, respectively. Only the glycosylated forms (glycosides) of each cardiotonic steroid under study are represented in Figure 1. The aglycones (Table 1) correspond to the compounds without the sugar moiety.

Of the five cardenolides analyzed, gitoxin appeared to be the least potent cardenolide in terms of growth inhibitory activity of human cancer cells (Table 1). In contrast, the two bufadienolides, gamabufotalin-rhamnoside and hellebrin, appeared to be much more potent than the cardenolides in terms of *in vitro* growth inhibition of human cancer cells (Table 1).

**Figure 3:**
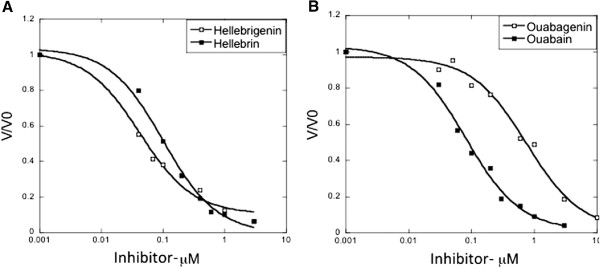
**Inhibition of purified human α1β1 Na**^**+**^**/K**^**+**^**-ATPase by the glycoside (closed circles) and aglycone (open circles) pairs of A: hellebrin/hellebrigenin and B: ouabain/ouabaigenin.** (panel **B**) Solid lines are the fitted curves for a one site fitted model (see Materials and Methods).The curves represent the averages of two experiments in duplicates.

As expected from the numerous data published in the literature, most cardiotonic steroid aglycones displayed weaker *in vitro* growth inhibition than the corresponding glycosides (Table 1). This was observed clearly for the cardenolides (ouabain / ouabagenin and digoxin / digoxigenin) and also for the bufadienolide pair gamabufotalin-rhamnoside / gamabufotalin (mean IC_50_ ± SEM: 9 ± 2 versus 26 ± 4 nM, p = 0.02). However, one clear exception was noticed with hellebrin and hellebrigenin (Table 1), for which the aglycone was not less effective than the glycoside. We observed a tendency for lower values of IC_50_ for hellebrigenin (mean IC_50_ ± SEM: 16 ± 5 nM) compared to hellebrin (mean IC_50_ ± SEM: 28 ± 7 nM), although the difference was not statistically significant (also see the data in Table 2 and Figure 3).

**Table 2:** **NaK alpha-subunit-binding affinity profiles for various cardenolides and bufadienolides (K**_**D**_ **± SEM)**

**Compounds**	**Affinity (nM)**			**Selectivity**	
	**α-1**	**α-2**	**α-3**	**α-1 / α-2**	**α-1 / α-3**
**Cardenolides**					
Ouabain	50 ± 10	70 ± 12	50 ± 7	0.7	1.0
Digoxin	104 ± 11	25 ± 4	27 ± 2	4.2	3.9
Uzarigenin-rhamnoside	90 ± 20	96 ± 7	122 ± 9	0.9	0.7
Uzarigenin	1,100 ± 120	883 ± 56	1,278 ± 142	1.3	0.9
Gitoxin	123 ± 8	85 ± 3	108 ± 17	1.5	1.1
Gitoxigenin	500 ± 90	1,180 ± 190	1,050 ± 100	0.4	0.5
**Bufadienolides**					
Gamabufotalin-rhamnoside	30 ± 7	36 ± 10	17 ± 2	0.8	1.8
Gamabufotalin	243 ± 48	247 ± 30	236 ± 14	1.0	1.0
Hellebrin	86 ± 18	218 ± 52	175 ± 20	0.4	0.5
Hellebrigenin	103 ± 19	198 ± 51	191 ± 23	0.5	0.5

*The data were obtained from three independent experiments.

*The data for uzarigenin, uzarigenin-rhamnoside, gitoxigenin and gitoxin are from Katz et al. [5] and are included here.

*Ouabain binding affinities for the three isoforms appear lower than analyzed previously [5]. The lower values in [5] are the result of full Scatchard analysis for ouabain which excludes all non-specific binding. The selectivity is the same even though the absolute values of K_D_ are higher. All the experiments were done in the same way, e.g. we have left all the values obtained in the displacement experiments.

Whether the genetic profiles (mutations) of the cell lines under study influence the cardiotonic steroid-mediated *in vitro* growth inhibition remains to be determined. The present study however reveals that p53 status does not influence cardiotonic steroid-mediated effects on *in vitro* growth rates of human cancer cells as detailed below.

A non-cancerous control cell line, i.e. NHDF fibroblasts, was used to analyze the *in vitro* growth inhibitory effects of gamabufotalin-rhamnoside / gamabufotalin and hellebrin / hellebrigenin. The data obtained were gamabufotalin-rhamnoside: 1,840 nM; gamabufotalin: > 10,000 nM; hellebrin: > 10,000 nM, and hellebrigenin: > 10,000 nM. Thus, these four compounds display weaker growth inhibitory effects in normal fibroblasts than in cancer cells, as already observed previously with other cardiotonic steroids [10,12].

All of the compounds described in Table 1 were also assayed for *in vitro* growth inhibitory activity in two mouse cancer cell lines: the CT26 colon cancer and the B16F10 melanoma cell lines. Except for gamabufotalin-rhamnoside, all of the compounds displayed IC_50_ growth inhibitory concentrations higher than 10,000 nM (data not shown). Rodent NaK shows a roughly 1,000-fold lower affinity for ouabain compared to human NaK (detected as Ki for inhibition of activity of 100 and 0.1 μM, respectively). This feature is caused by two mutations (human-rat Q117R and N128D) in the extracellular loop between TM1 and TM2 in murine α1 compared to human α1, which accounts for the low ouabain-binding affinity of the rodent pump and the approximately 1,000-fold weaker sensitivity of murine cancer cells to the growth inhibitory effects of cardiotonic steroids [2]. Yang et al. [27] demonstrated that the relative lack of the NaK α3 subunit in rodent cancer cells may also account for their unresponsiveness to cardiotonic steroids, but Lin et al. [28] recently emphasized the importance of the NaK α1 subunit in tumor growth and cancer cell survival. Gamabufotalin-rhamnoside displayed IC_50_ concentrations of 0.9 μM in CT26 colon cancer cells and 0.7 μM in B16F10 melanoma cells, which are concentrations that are about one hundred times lower than those observed in human cancer cells (Table 1).

### Hellebrin and hellebrigenin overcome apoptosis-resistance in cancer cells

Of the eight cancer cell lines reported in Table 1, we have experimental evidence of various levels of resistance of A549 NSCLC cells [29,30], U373 GBM cells [10,31] and SKMEL-28 melanoma cells [32] to pro-apoptotic stimuli. In the same manner, we have experimental evidence of sensitivity to pro-apoptotic stimuli for Hs683 oligodendroglioma cells [31,33], and MCF-7 breast cancer [34] and PC-3 prostate cancer [34] cells. The data in Table 1 indicate that the various cardenolides and bufadienolides used in this study (including hellebrigenin) display similar *in vitro* growth inhibitory activity in cancer cells that display sensitivity versus those that display certain levels of resistance to pro-apoptotic stimuli.

### Hellebrin and hellebrigenin overcome MDR resistance in cancer cells

**Figure 4:**
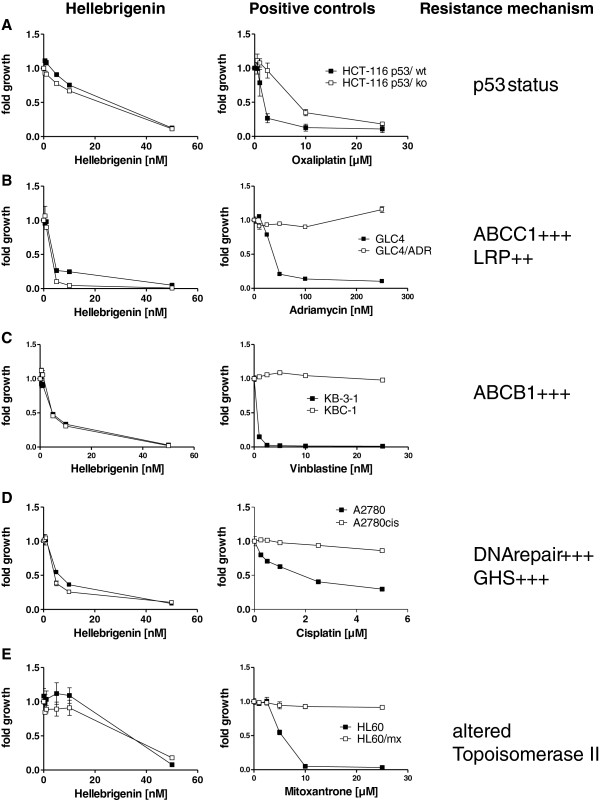
**Impact of different MDR mechanisms on the cytotoxicity of hellebrigenin.** The different drug-resistant cancer cell models (as indicated) were treated for 72 hours with hellebrigenin (left panels) and with the respective drugs affected by the indicated resistance mechanisms as positive controls (right panels). Viability was determined using a 3-(4,5-dimethythiazol-2-yl)-2,5-diphenyltetrazolium bromide (MTT) colorimetric assay. Each assay was carried out in six replicates. The various cell lines used are **A:** the colon carcinoma cell line HCT-116 p53/wt and the p53 knock-out cell line HCT-116 p53/ko; **B:** the small cell lung carcinoma cell line GLC-4 and the ABCC1- and LRP-overexpressing subline GLC-4/ADR; **C:** the epidermal carcinoma-derived cell line KB-3-1 and the ABCB1-overexpressing subline KB-C-1; **D:** the ovarian carcinoma cell line A2780 and the cisplatin resistant subline A2780cis; **E:** the promyelocytic leukemia cell line HL60 and the mitoxantrone resistant subline HL60/mx. Cells were not starved during the experiments.

Many ATP-binding cassette (ABC) transporters are implicated in the MDR phenotypes of cancer cells [35,36]. Therefore, hellebrin and hellebrigenin were assayed in various ABC models of MDR cancer cells, as detailed in Figure 4.

First, Figure 4A confirms the data from Table 1. Indeed, hellebrigenin and hellebrin (data not shown) display similar *in vitro* growth inhibitory activity in p53 wild-type versus p53KO human HCT-116 colon cancer cells.

Hellebrigenin and hellebrin (data not shown) are equally active in several MDR cancer cell models with chemotherapy resistance based on the overexpression of ABC-transporters and/or altered glutathione metabolism. Thus, the strongly ABCC1- and LRP-overexpressing small cell lung cancer GLC4/ADR (Figure 4B) and the colchicine-selected, ABCB1-overexpressing, HeLa cell subclone KB-C-1, were equally sensitive against hellebrigenin as their parental cell lines (Figure 4C). Additionally, neither cisplatin resistance based on altered glutathione metabolism and enhanced DNA repair (Figure 4D) nor mitoxantrone resistance caused by alterations of topoisomerase II (Figure 4E) conferred reduced sensitivity to hellebrigenin.

### Hellebrin and hellebrigenin are cytotoxic compounds

**Figure 5:**
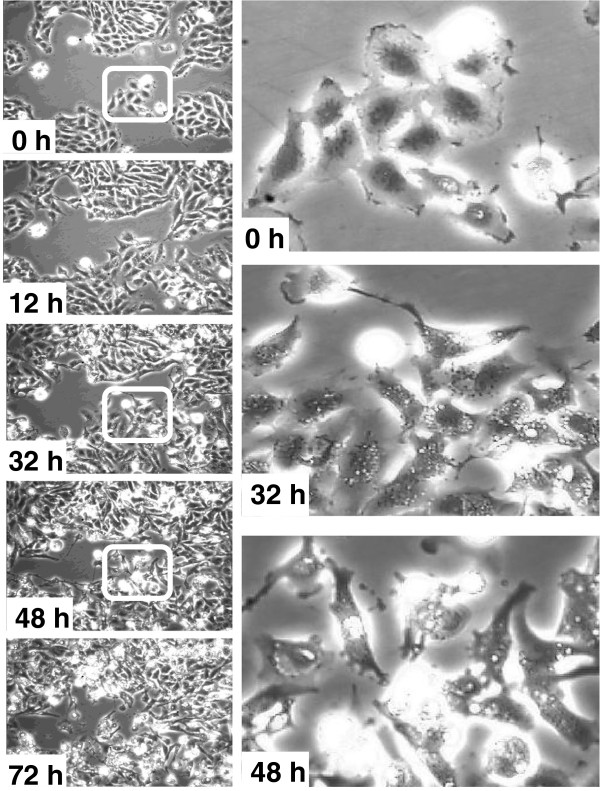
**Computer-assisted phase-contrast microscopy (quantitative videomicroscopy) illustrations of human U373 glioblastoma cells treated with 10 nM hellebrigenin for 72 h.** The squares on the left-handed panel of images (0 – 32 – 48 h) highlight the morphological aspects of the U373 GBM cells on the right-handed images at a higher magnification, with the appearance of marked vacuolization processes. Cells were not starved during the experiments. The images provided here are representative images obtained from triplicates in each experimental condition.

Computer-assisted phase contrast microscopy (quantitative videomicroscopy) analyses were used to morphologically visualize the effects induced by hellebrin (data not shown) and hellebrigenin (Figure 5) in human U373 GBM cells. Hellebrigenin was assayed in U373 GBM cells at the IC_50_ growth inhibitory concentration of 10 nM, which was determined by the MTT colorimetric assay (Table 1). Numerous cytoplasmic vacuoles were observed after 12 h of U373 cell treatment with 10 nM hellebrigenin, and this effect was sustained up to 32–40 h after treatment (Figure 5). Then, between 45–50 h, U373 GBM cells began to die, and, in accordance with the MTT assay-related data, approximately 50% of the U373 GBM cells died after 72 h of treatment with 10 nM hellebrigenin (Figure 5). These morphological analyses revealed that hellebrigenin is a cytotoxic compound and not a cytostatic one. Similar results were observed with respect to hellebrin (data not shown).

Previous experiments that were carried out with the 19-hydroxy-2″-oxovoruscharin cardenolide pointed to vacuolization processes in human A549 NSCLC cells [30] and U373 GBM cells [10] that were similar to the vacuolization displayed by hellebrigenin in human U373 GBM cells (Figure 5). These vacuolization processes induced by 19-hydroxy-2″-oxovoruscharin led to sustained and irreversible autophagy-related cell death in U373 GBM cells [10] and to lysosomal membrane permeabilization-related cell death in A549 NSCLC cells [30].

### Low concentrations of bufadienolides inhibit oxidative metabolism of human HT-29 colon cancer cells

**Figure 6:**
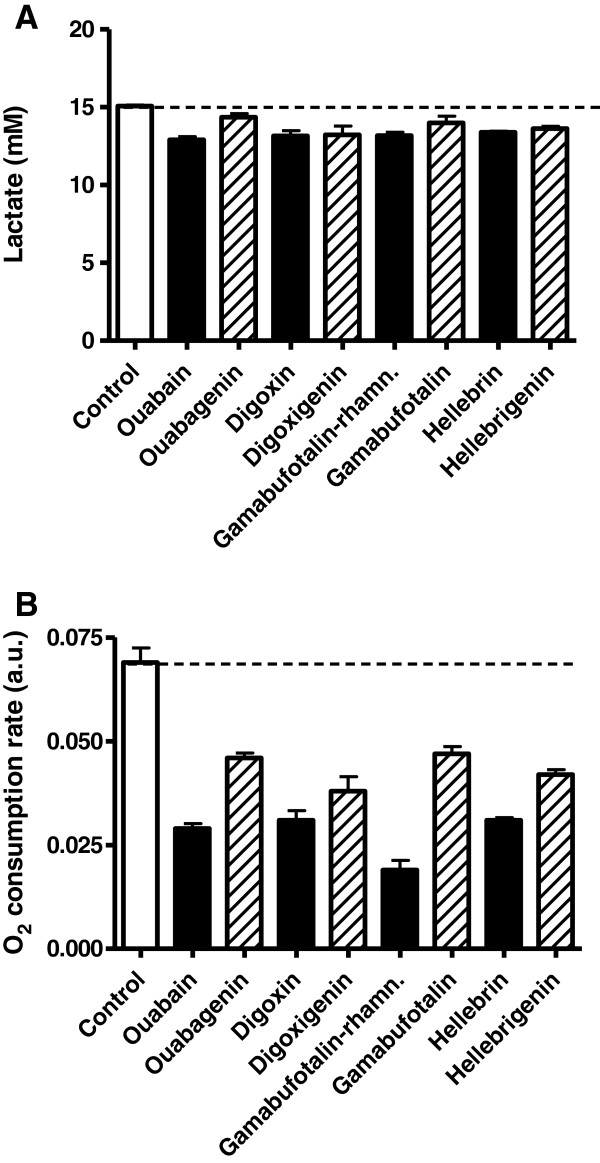
**Metabolic profiling of human HT29 colon cancer cells treated with cardiotonic steroids. A:** Extracellular lactate concentrations and **B:** O_2_ consumption rates in HT29 cancer cells treated with the indicated compounds at their IC_50_*in vitro* growth inhibitory concentrations (see Table 1). The data (mean ± SEM from triplicates) are expressed in mM lactate accumulated in the extracellular medium after 24 hours and arbitrary units derived from time-resolved fluorescent signal.min^-1^.

The vacuolization processes reported in the previous section with respect to 19-hydroxy-2″-oxovoruscharin cardenolide in human A549 NSCLC [30] and U373 GBM [10] were paralleled by marked decreases in intracellular ATP concentration ([ATP]_i_) in these cells [10,12], while much weaker effects were observed in normal cells [10,12]. Therefore, we analyzed the effects induced by four cardenolides and four bufadienolides (including hellebrin and hellebrigenin) on glycolysis and O_2_ consumption rates in human HT-29 colon cancer cells. The preliminary data (not shown) indicated that the HT-29 cell line exhibited both glycolytic (glucose to lactate) and oxidative (glucose to CO_2_) metabolism. While cardenolides and bufadienolides did not alter the glycolytic flux as determined by the measurements of lactate release in the extracellular medium (Figure 6A), each compound significantly (P < 0.01) influenced cell respiration (Figure 6B). The O_2_ consumption rate was significantly reduced following treatment with digoxin, ouabain, hellebrin and gamabufotalin-rhamnoside at the IC_50_*in vitro* growth inhibition of each compound (as determined in the MTT assay). A trend to a higher activity of the glycosides versus aglycones was also observed. Of note, these measurements were obtained after 24 h (Figure 6), a delay for which the confounding effects of cell death could be excluded (Figure 4).

### Characterization of the binding affinity and inhibitory potential of cardiotonic steroids for human NaK subunits

Table 2 details the binding affinity and selectivity of various cardenolides and bufadienolides for the human α1β1, α2β1 and α3β1 NaK complexes measured as competitive inhibition for ^3^H-ouabain-binding on yeast membranes expressing the different isoforms [5,26]. These data confirm the recent observation that digoxin displays 3- to 4-fold higher binding activity for human α2β1 and α3β1 than for α1β1, while ouabain displays similar binding affinities for the three NaK complexes (Table 2) [5]. The data in Table 2 also show that gamabufotalin-rhamnoside shows a much higher affinity compared to gamabufotalin (aglycone), but no subunit selectivity of the aglycone. These data are compatible with the conclusion that the sugar determines the isoform selectivity [5]. The data from Table 2 point to an unusual feature of hellebrin- and hellebrigenin-binding, which is that there is no difference in affinity between the glycoside and aglycone forms. There also appears to be a moderate selectivity for the α1 compared to the α2 and α3 subunits of both hellebrin and hellebrigenin, but some of the differences are not significant. This unusual feature of hellebrin- and hellebrigenin-binding detected in the ^3^H-ouabain displacement assays was confirmed in assays of the inhibition of NaK activity of purified human α1β1 (Figure 3). Strikingly, hellebrigenin was more effective than hellebrin in this inhibition assay (Ki 46 ± 6 and 103 ± 7 nM, respectively), whereas inhibition by ouabain and ouabagenin showed the characteristic effect of a lower Ki for the glycoside (Ki 97 ± 5 and 721 ± 70 nM, respectively). A comparison of the inhibition of NaK activity of all of the purified complexes showed no selectivity for either hellebrin or hellebrigenin, while hellebrin and hellebrigenin nevertheless displayed a two times higher affinity for α1β1 than for α2β1 or α3β1 complexes (Table 2).

Figure 2A indicates a strong linear correlation between the *in vitro* IC_50_ growth inhibitory concentrations of various cardiotonic steroids (ouabain, digoxin, hellebrin, hellebrigenin, gamabufotalin rhamnoside, and oleandrin) and the Ki for inhibition of the purified human NaK α1β1 complex (IC_0.5_ = 0.354 x K_i_ - 0.170; r = 0.98). By contrast, Figure 2B also reveals that there is no correlation between these IC_50_ growth inhibitory concentrations and the levels of α1 expression (determined at the level of mRNA by means of quantitative RT-PCR). Together, these observations imply that the IC_50_ values for growth inhibition by the different compounds are a consequence of binding to and inhibition of the pump.

## Discussion

The Na/K pump maintains the concentration gradients of Na^+^ and K^+^ ions across the surface membrane of animal cells [6], and a substantial amount of surface-expressed NaK, especially in cancer cells, has been suggested to function as non-canonical cardiotonic steroid-binding receptors [7] that activate multiple signaling cascades [8-11].

Multiplex gene expression analysis demonstrated a decade ago that various cardiotonic steroids inhibit prostate target genes [37]. Several studies have demonstrated that various cardiotonic steroids are able to sensitize apoptosis-resistant cancer cells to pro-apoptotic stimuli [38-40] and directly induce apoptosis in lymphoma [41] and leukemia [42] cells. Cardiotonic steroids can also induce cancer cell death through Src- or MAPK-mediated inhibition of p53 expression [43], the inhibition of general protein synthesis [44], the inhibition of HIF-1*a* synthesis [45], sustained and irreversible autophagy [10,46] and lysosomal membrane permeabilization [30].

Some cardiotonic steroids are also able to overcome the MDR phenotype. Indeed, while ouabain activates the MDR phenotype [47], 19-hydroxy-2″-oxovoruscharine [48] and the hellebrin / hellebrigenin pair (Figure 4) display similar and marked anticancer activity in chemosensitive versus MDR cancer cells.

Thus, cardiotonic steroids display pleotropic anticancer effects, and we recently reviewed all of the patents filed in this field, along with their potential applications in oncology [49].

We previously reported that the 19-hydroxy-2″-oxovoruscharin cardenolide induced marked decreases in [ATP]_i_ in various cancer cell types, while much weaker effects were observed in normal cells [10,12]. This observation was confirmed in the present study with respect to gamabufotalin-rhamnoside / gamabufotalin and hellebrin / hellebrigenin. We provide evidence in this study that the previously observed drop in intracellular ATP in tumor cells exposed to cardiotonic steroids is unlikely to arise from an alteration in the glycolytic flux. The extent of glucose-to-lactate conversion remained unaltered in HT29 colon cancer cells treated either with cardenolides or bufadienolides. Instead, we found a dramatic reduction in the oxygen consumption rate in cells treated with cardenolides and bufadienolides, reflecting a direct impact on the mitochondrial oxidative phosphorylation. While these effects on cell respiration were quite similar for each compound tested, it should be emphasized that bufadienolides (glycosylated or not) were used at a concentration in the low nanomolar range (10–30 nM), while cardenolides were used at concentrations ~3-fold (ouabain), 10-fold (digoxine) and 150-fold higher (ouabagenin, digoxigenin). This observation again supports the specific profile of bufadienolides and identifies tumor cell oxidative metabolism as a major target of these drugs.

The glycosylation patterns of cardiotonic steroids markedly influence their anticancer activity profiles. For example, Langenhan et al. [50] demonstrated that the glycorandomization of digitoxin leads to analogs that display significantly enhanced anticancer activity and tumor specificity when compared to digitoxin. In addition, changes in NaK expression dictate the growth regulatory effects of ouabain on cells [51]. In the current study, the quantitative determination of α1, α2 and α3 subunits at the mRNA level in the various cancer cell lines that were used clearly indicated that all of the cell lines expressed the α1 subunit, though in a heterogeneous manner (Figure 2B), but did not express the α2 or α3 subunits (or they expressed the α3 subunit in very low amounts) (data not shown).

Table 2 includes previously published data [5] and shows that ouabain could display weak selectivity for the α1 subunit, while digoxin shows a 3- to 4-fold selectivity for α2/α3. The other cardenolides, uzarigenin and gitoxin, do not show isoform selectivity, although the glycosides have much higher affinities compared to the aglycones. For the bufadienolide gamabufotalin / gamabufotalin-rhamnoside pair, the rhamnoside appears to show marginal selectivity for α1 when compared to α2 but not to α3. There is also a clear difference in that the glycoside shows a much higher affinity than the aglycone (Table 2). By contrast, the hellebrin / hellebrigenin pair is anomalous in that the glycoside does not show a higher affinity for ^3^H-ouabain displacement compared to the aglycone, and in NaK inhibition assays the aglycone is even somewhat superior to the glycoside (Figure 3). Of all the cardiotonic steroids analyzed, the hellebrin / hellebrigenin pair displayed the highest selectivity for the NaK α1 subunit at an approximately 2 times higher affinity for the α1 than for the α2 and α3 subunits (Table 2).

The features of binding and inhibition of the human α1β1 complex of the different cardiac glycosides are reflected in the IC_50_ concentrations for growth inhibition. A positive correlation was shown between the IC_50_ for growth inhibition and Ki for inhibition of the purified α1β1 complex, and there is no evidence for a pattern typical of inhibition of α2 or α3 (Figure 2A). For example, digoxin shows a higher Ki and IC_50_ compared to ouabain. This is typical for α1, whereas digoxin should show a lower Ki and IC_50_ than ouabain in the cases of α2 and α3 subunits [5]. The data for the bufadienolides are consistent with these conclusions, especially the conclusion that a lower Ki for α1β1 is associated with a lower IC_50_ for growth inhibition. The anomaly in binding and inhibition occurs with the hellebrin / hellebrigenin pair, a feature that may be related to the fact that the second sugar in hellebrin is glucose. In systematic studies, it has been found that glucose is not an optimal glycoside derivative for the binding or inhibition of renal NaK α1β1 [52,53]. It has also been previously shown that the relative effects of glycoside and aglycone on Ki for inhibition of the renal NaK vary markedly in the function of different cardiac steroids [54]. The parallel behavior between binding and inhibition of α1β1 versus cancer cell growth inhibition is also observed for the hellebrin / hellebrigenin pair. The findings that mouse cancer cells display high IC_50_ values for growth inhibition by different cardiac glycosides and that the growth effects of gamabufotalin-rhamnoside are in the μM range (compared to the nM range for the human cancer cells) also demonstrate the association of cancer cell growth inhibition and inhibition of the low affinity cardiac glycoside-binding rodent α1β1 complex. The gamabufotalin-rhamnoside is therefore a useful tool to check this association in rodent cells because even a “low affinity” effect is in the μM concentration range.

## Conclusions

The clear correlation between the IC_50_ growth inhibitory concentration and Ki for inhibition of NaK activity of purified human α1β1 suggests that the inhibition of α1β1 is the first step in the cancer growth inhibition effects of cardiac glycosides, provided that NaK α3β1 is not more present *in vitro* (as in the case of the current study). This feature is also true for some cancer types that overexpress the NaK α1β1 but not the α3β1 complex, such as gliomas [10], melanomas [16], NSCLCs [12] and renal cell carcinomas [17]. Anomalies in the expected pattern of behavior of the glycoside and aglycone, such as in the case of hellebrigenin and hellebrin, further strengthen this correlation. Thus, hellebrigenin, with its free C3 position, might be derivatized into novel analogs to increase the selectivity for the α1 subunit and make these original optimized hellebrigenin analogs novel weapons to combat those gliomas, melanomas, NSCLCs and renal cell carcinomas that overexpress this α1 subunit. While the mechanism of the cytotoxic effects of cardiac glycosides is not yet entirely deciphered [2,4,55], the current study highlights a common feature for all of the cardiotonic steroids we analyzed, including cardenolides and bufadienolides, which is a dramatic reduction in the oxygen consumption rate in cardenolide- and bufadienolide-treated cells. These data suggest a direct impact on mitochondrial oxidative phosphorylation. In this respect, it is also interesting that the cardiotoxicity of ouabain appears to be associated with the disruption of mitochondrial Ca^2+^ handling and NAD/NADH ratios [56-58]. This feature could explain why i) cardiotonic steroids are more toxic with respect to cancer than normal cells and ii) certain cardiotonic steroids are able to overcome the intrinsic resistance of cancer cells to pro-apoptotic stimuli without activating the MDR phenotype, even if these cardiotonic steroids are cytotoxic, such as the hellebrin / hellebrigenin pair (the current study) or 19-hydroxy-2″-oxovoruscharine [48].

## Abbreviations

ATCC: American type culture collection; DSMZ: Deutsche Sammlung von Mikroorganismen and Zellkulturen; ECACC: European Collection of Cell Culture; GBM: Glioblastoma; GGR: Global growth ratio; MDR: Multidrug resistance; MTT: 3-(4,5-dimethylthiazol-2-yl)-2,5-diphenyltetrazolium bromide; NaK: Na^+^/K^+^-ATPase; NSCLC: Non-small-cell lung carcinoma; QVM: Quantitative videomicroscopy.

## Competing interests

None of the listed authors have competing interests related to the publication of this manuscript.

## Authors’ contributions

AE, BK, WB, OF, SK and RK conceived of the study and designed the assays. LMYB performed *in vitro* growth inhibition measurements and quantitative videomicroscopy analyses. WM performed the MDR assays. AC, DMT, MZ and EU performed the analytical chemistry analyses and compound purification. AK and EA performed the NaK binding studies. AE, BK, WB, OF, SK and RK wrote and edited the manuscript. All authors read and approved the final manuscript.
